# High Blood Levels of Cyclophilin A Increased Susceptibility to Ulcerative Colitis in a Transgenic Mouse Model

**DOI:** 10.3390/ijms262412068

**Published:** 2025-12-15

**Authors:** Iuliia P. Baikova, Leonid A. Ilchuk, Marina V. Kubekina, Anastasiia A. Kalinina, Ludmila M. Khromykh, Yulia D. Okulova, Natalia G. Pavlenko, Diana S. Korshunova, Eugenii N. Korshunov, Alexandra V. Bruter, Yulia Yu. Silaeva

**Affiliations:** 1 Institute of Gene Biology, Russian Academy of Sciences, Vavilov St., 34/5, Moscow 119334, Russia; baykjulia@gmail.com (I.P.B.); lechuk12@gmail.com (L.A.I.); marykumy@gmail.com (M.V.K.); ul.okulova@gmail.com (Y.D.O.); npavlenko19@yandex.ru (N.G.P.); diana13021993@gmail.com (D.S.K.); jack.des.kollord@gmail.com (E.N.K.); aleabruter@gmail.com (A.V.B.); 2 N.N. Blokhin National Medical Research Center of Oncology, Ministry of Health of the Russian Federation, 24 Kashirskoe sh., Moscow 115478, Russia; aakalinina89@gmail.com (A.A.K.); lkhromykh@list.ru (L.M.K.)

**Keywords:** colitis, inflammation, animal model, cyclophilin A

## Abstract

Mouse models of human autoimmune diseases and inflammation are a challenging field because of the relatively low homology between the human and mouse immune systems. At the same time, inflammation plays a significant role in the pathogenesis of many diseases, strongly impacting quality of life and mortality. Cyclophilin A (CypA) is a pro-inflammatory factor, the ligand of immunosuppressive cyclosporin A, which mediates inflammation through multiple signaling pathways. Here, we describe a novel transgenic mouse model with Cre-dependent expression of the *hPPIA* gene in vascular endothelium and secretion of CypA into the bloodstream, which shows elevated blood levels of CypA upon activation. Being mostly asymptomatic under standard conditions, these mice exhibited more severe inflammation when provided with 3% dextran sulfate sodium solution instead of drinking water for 7 days. Inflammation symptoms precisely resembled those of ulcerative colitis and included deterioration of the colon crypts alongside the relatively normal duodenum. These results show that the elevated blood level of CypA enhances induced inflammation but does not cause inflammation by itself, suggesting its role in pro-inflammatory positive feedback loops and making CypA a suitable anti-inflammatory target. Moreover, our mouse strain is an applicable colitis model and can be used further in emerging inflammation research and testing anti-CypA targeted therapy.

## 1. Introduction

Being an essential part of innate immunity, inflammation is indispensable for defense against different pathogens and wound healing. Despite this, inflammation can be disadvantageous if it becomes chronic or is triggered by an inappropriate nonpathogenic cause.

Chronic inflammation contributes to the development of several diseases with significant social impact, including, first of all, various cancers and autoimmune diseases. According to some estimates, autoimmune diseases affect as much as 10% of certain populations, and their incidence is increasing [1].

Inflammatory bowel diseases (IBDs), including Crohn’s disease (CD) and ulcerative colitis (UC), may not be among the most dangerous auto-inflammatory diseases; however, they are among the most widespread (up to 1% of the population in Western countries), can significantly affect quality of life, and are widely recognized as cancer harbingers [2,3].

Chronic inflammation can be regarded as a result of the action of a positive feedback loop that is active long after an initial trigger was withdrawn [4,5,6,7]. From this point of view, multiple components of the auto-inflammatory cascade can be targeted to mitigate chronic inflammation. However, as of today, the main treatment approaches remain nonspecific and can cause general immunosuppression and serious side effects. This work focuses on one such molecule—Cyclophilin A (CypA), the product of the *PPIA* gene, which appears to be a promising drug target.

CypA, a multifunctional secreted pro-inflammatory protein, exhibits pleiotropic immunomodulatory effects and regulates production and functions of other pro-inflammatory mediators—interferon-γ (IFNγ), tumor necrosis factor-α (TNFα), interleukin (IL)-6, IL-8, and IL-1β [8,9,10]. It has gained certain attention as a factor associated with counterproductive inflammation in cardiac and cardiovascular disease, diabetes, neurodegeneration, and cancers [11]. Elevated CypA levels are associated [11] with a more severe disease progression both in animal models and in patients [12,13,14].

High blood levels of secreted CypA were detected in UC, but not CD patients, and it was suggested as an additional serological biomarker for IBD [15,16], although it still has not been included in conventional clinical test panels [17]. Furthermore, it was shown to contribute greatly to IBD pathogenesis itself [16]. Elevated serum concentrations of CypA enhanced the expression of matrix metalloproteinase 9 and TNFα, thus promoting IBD development, namely, UC [16]. Importantly, the expression of CD147, the main receptor of extracellular CypA, was found to be upregulated in the intestinal mucosa of patients with IBD [18]. The interaction between secreted CypA and CD147 was proposed as the main mechanism involved in IBD pathogenesis, which triggered the production of other pro-inflammatory mediators and aggravated inflammation [16].

Despite the well-established roles of secreted CypA in the pathogenesis of inflammation-associated diseases, and IBD in particular, its potential functions as a trigger of such pathological conditions require further elucidation. It remains unclear whether CypA, as a key factor in chronic inflammation, could enhance susceptibility to inflammation-associated diseases.

To further study the role and the mechanisms of CypA action in inflammation-associated diseases, we established a transgenic mouse strain with Cre recombinase-inducible expression of the human *PPIA* gene encoding CypA. We chose the inducible expression because our previous work [19] demonstrated that CypA overexpression in embryos can impact pregnancy progression and embryo development. At the same time, tissue-specific expression permitted us to dissect the phenotypes arising in different organs and systems. Previous experiments with CypA-overexpressing and knockout mice demonstrated a dual and context-dependent role for CypA. In protective contexts, CypA reduces susceptibility to infection [20] and nephrotoxicity associated with immunosuppressive treatment [21]. Conversely, it promotes harmful effects, including hyperplasia and inflammation in carotid ligation models [22], cardiac hypertrophy in ApoE mice [23], and ischemia–reperfusion injury in a myocardium infarct model [24].

Here, we focus on the study of intestinal autoinflammation in mice with human CypA secreted in the bloodstream and show that under normal conditions, these mice are generally asymptomatic; however, when challenged with low doses of dextran sulfate sodium (DSS), they exhibit much more severe symptoms than control mice. We suggest that these mice can serve not only as a model to study the mechanisms of inflammation and CypA-related mechanisms but also as a tool to study emerging CypA inhibitors [25], as the human *PPIA* gene is expressed.

## 2. Results

### 2.1. Transgene Generation and Validation

**Figure 1:**
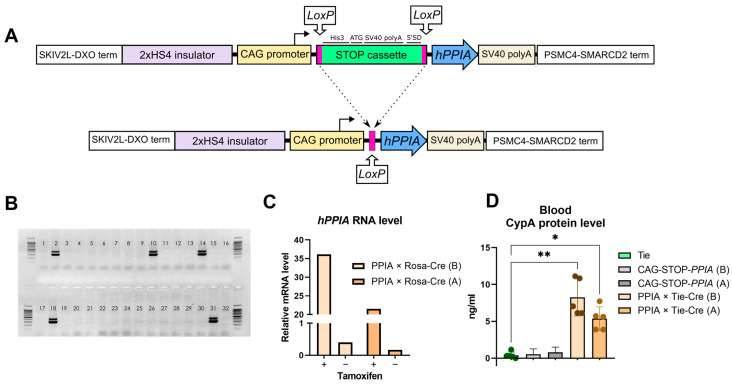
CAG-STOP-*PPIA* mice generation and verification. (**A**) The gene construct used for transgenesis, the CAG promoter, is constitutive and unidirectional. STOP-cassette includes His3—yeast His3 C-terminal gene region, ATG—false start codon repeats, SV40 polyA—Simian virus 40 polyadenylation signal, and 5′SD—splice donor site. (**B**) Genotyping of F_0_ mice. DNA from samples numbered 2, 10 (substrain A), 14, and 18 (substrain B) contains the transgene. Lane 31—positive control (the initial plasmid); lane 32—negative control (wild type). (**C**) qPCR for *hPPIA* expression in CAG-STOP-*PPIA*×R26-Cre-ER^T2^ (PPIA×Rosa-Cre) mice for both A and B substrains. (**D**) The CypA concentration in the blood of CAG-STOP-*PPIA*×Tie2-Cre (PPIA×Tie-Cre) for both A and B substrains; Kruskal–Wallis test with Dunn’s post hoc test. * *p* < 0.05, ** *p* < 0.021.

Two CAG-STOP-*PPIA* substrains (A and B) were established from four PCR-positive founders derived from microinjected zygotes (Figure 1A,B). After four backcrosses to C57BL/6, validated transgenic mice were crossed with R26-Cre-ER^T2^ mice, producing PPIA×Rosa-Cre offspring. Upon tamoxifen administration, the PPIA×Rosa-Cre mice showed >100-fold *hPPIA* induction relative to the control, with the B substrain showing 2-fold higher expression than A (Figure 1C) measured in blood cells. Subsequent validation in vascular endothelium-specific PPIA×Tie-Cre mice revealed dose-dependent CypA in serum, with comparable levels between substrains (Figure 1D).

### 2.2. PPIA×Tie-Cre Mice Exhibited More Severe Symptoms of Ulcerative Colitis When Exposed to DSS

Preliminary dose–response experiments identified single-round 3% DSS as an appropriate challenge scheme—lower doses yielded only minor symptoms, whereas higher doses resulted in substantial mortality (Table 1).

**Table 1:** Treatment regimens, disease activity index (DAI) scores (Table 2), and histological pathology in dextran sulphate sodium (DSS)-induced colitis in wild-type animals.

Treatment	Scheme	Total/Deceased	DAI	Histological Pathology
Water	7W ^1^	3/0	0	None
1% DSS	7D + 7W	5/0	0	Duodenum: none Colon: local infiltration of l.p. ^3^
	7D + 7W + 7D	5/0	1 = 1 + 0 + 0 + 0 ^2^	Duodenum: slight l.p. swelling Colon: local infiltration of l.p. Crypt structure unaltered
Water	7W	3/0	0	None
3% DSS	7D + 7W	5/0	3 = 1 + 1.5 + 0.5 + 0	Duodenum: low Paneth and goblet cell counts, swelling of l.p Colon: chronic inflammation, l.p. infiltration, ⅓ crypts lost
	7D + 7W + 7D	5/3	6 = 2 + 2.5 + 1 + 0.5	Duodenum: low Paneth and goblet cell counts, swelling of l.p Colon: acute inflammation, extensive l.p. infiltration, 2/3 crypts lost
Water	7W	3/0	0	None
5% DSS	7D + 7W	5/3	8 = 3 + 3 + 1 + 1	Duodenum: low Paneth and goblet cell counts, swelling of l.p. Colon: acute inflammation, full loss of crypts
	7D + 7W + 7D	5/5	-	-

^1^ Regimens are presented as consecutive (+) modes of treatment by water (W) or DSS (D) for 7 days. ^2^ DAI is a sum of scores described in Table 2, presented in the following summation order: weight loss, stool consistency, rectal bleeding, and anal prolapse group average scores. ^3^ l.p. stands for lamina propria.

Fifteen control Tie-Cre and 15 PPIA×Tie-Cre (substrain A) mice were enrolled in the experiment: 8 mice of each genotype were treated with DSS, whereas 7 formed the control group.

**Figure 2:**
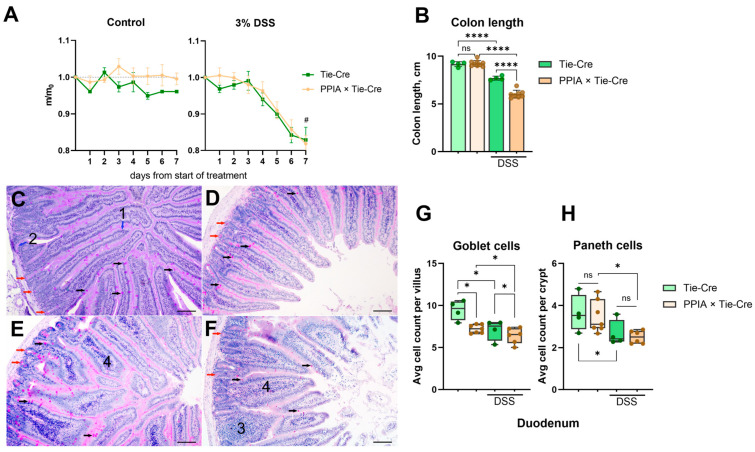
General condition and changes in the duodenum of dextran sulphate sodium (DSS)-treated mice. (**A**) Weight loss after DSS treatment; mean ± 95% CI. (**B**) Colon shortening after DSS treatment; mean ± SD; one-way ANOVA with post hoc test. (**C**–**F**) Cross-sections of the duodenum; periodic acid Schiff reaction. (**C**)—Tie-Cre, control group; (**D**)—PPIA×Tie-Cre, control group; (**E**)—Tie-Cre, DSS; (**F**)—PPIA×Tie-Cre, DSS. 1—villus. 2—crypt. 3—region of lymphoplasmacytic infiltration. 4—swelling of the lamina propria. Black arrows—goblet cells; red arrows—Paneth cells. Blue arrows denote structures with corresponding digits. Scale bar: 100 µm. (**G**,**H**) The mean number of goblet cells per villus and Paneth cells per crypt in the duodenum; two-way ANOVA with Šídák’s post hoc test. * *p* < 0.05, **** *p* < 0.0001, ns or # *p* > 0.05.

According to previously published data, DSS treatment causes weight loss prior to other symptoms [26]. We traced mouse weight in the experimental and control groups and registered an equal weight decline in all DSS-treated groups, independent of genotype (Figure 2A). Notably, the DSS-treated mice also displayed reduced food and water consumption and developed bloody diarrhea. These results confirm that DSS acts as expected [26]. Gastrointestinal symptoms became apparent on the third day in the PPIA×Tie-Cre group and on the fifth day in the Tie-Cre group. The symptoms in the PPIA×Tie-Cre animals were also more severe, and two out of eight DSS-treated PPIA×Tie-Cre mice died on the fourth day of treatment.

**Table 2:** Disease activity index (DAI) score criteria. Adopted from [27].

Score	Weight Loss	Stool Consistency	Rectal Bleeding	Anal Prolapse
0	loss < 1%	Separate hard lumps	No blood in stool	Not visible
1	1% ≤ loss < 5%	Sausage-like with cracks	Blood in stool	Externally visible
2	5% ≤ loss < 10%	Soft blobs		
3	10% ≤ loss <15%	Watery		
4	loss ≥ 15%			

After 14 days of treatment, the mice were sacrificed, and changes in their intestine were evaluated. First, we measured the length of the colons, as colon shortening is an important macroscopic marker of intestinal inflammation [26]. We observed that without DSS treatment, all genotype groups had equal colon length, indicating the absence of a severe phenotype in the untreated PPIA×Tie-Cre mice. At the same time, colon length shortened significantly (*p* < 0.0002) in all groups upon DSS treatment (Figure 2B), and in the PPIA × Tie-Cre group, the shrinkage was significantly (*p* < 0.0002) more pronounced than in the Tie-Cre group. This finding indicates that at the macroscopic level, DSS causes certain damage in both groups, while in combination with the high blood level of CypA, the damage is heavier.

Next, we performed a histological analysis of the duodenum and colon. In proper colitis experimental models, the colon should be severely impacted, while the duodenum should be unaffected or impacted only moderately. Before DSS treatment, all groups had similar epithelium morphology in the duodenum (Figure 2C,D) with a tendency (*p* < 0.05) of a decrease in the goblet cell number in the PPIA×Tie-Cre mice, which is a symptom of mild inflammation (Figure 2G). Staining of duodenum sections also revealed only mild changes in both groups after DSS treatment (Figure 2E,F). Epithelium morphology remained normal, and only a slight decrease in Paneth cells (in the PPIA×Tie-Cre group) and goblet cells (in both groups) was detected (Figure 2E–H).

**Figure 3:**
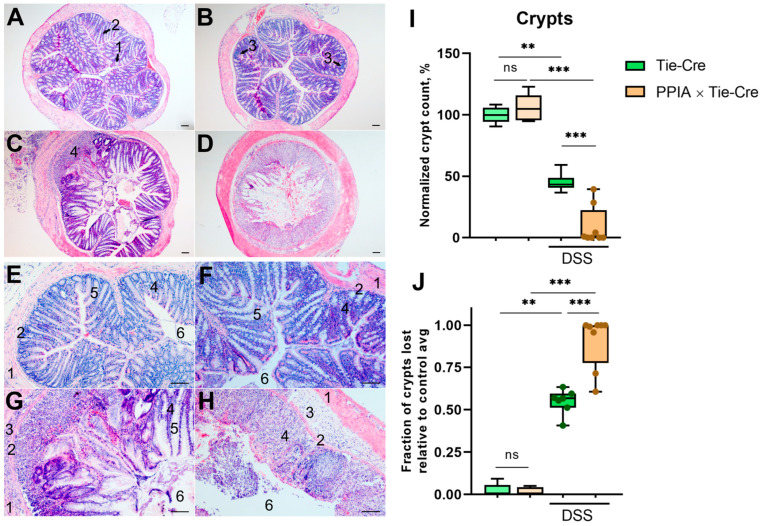
Pathomorphological changes in the colon of dextran sulphate sodium (DSS)-treated mice. (**A**–**D**) Cross-sections of the colon; hematoxylin–eosin staining at 4× magnification; 1—goblet cells of the crypt; 2—lamina propria; 3—local infiltration of the lamina propria; 4—focus of complete destruction of crypts. (**E**–**H**) Cross-sections of the colon, hematoxylin–eosin staining at 10× magnification; 1—muscularis externa; 2—muscularis mucosae; 3—submucosa; 4—lamina propria; 5—intestinal crypts; 6—lumen. (**A**,**E**)—Tie-Cre, control group; (**B**,**F**)—PPIA×Tie-Cre, control group; (**C**,**G)**—Tie-Cre, DSS, (**D**,**H)**—PPIA×Tie-Cre, DSS. Scale bars: 100 µm. (**I**,**J**) Quantification of loss of crypts; relative mean per whole section in n = 5–7 random cross-sections per mouse. The number of crypts in the control Tie-Cre group was taken as 100%. Exact Mann–Whitney test. ** *p* < 0.0021, *** *p* < 0.0002, ns *p* > 0.05 adjusted.

In the colon, however, we detected an entirely different pattern, as expected based on the colon length measurement. Despite no significant anomalies being registered in the PPIA×Tie-Cre colons before DSS treatment, prominent, locally even total, shortening of the crypts took place in the PPIA×Tie-Cre colons upon DSS treatment, while the colons in the control group were significantly less damaged (Figure 3A–H). The count of the crypts proves that there is no difference between the Tie-Cre and PPIA×Tie-Cre mice under normal conditions and that in both groups, the number of crypts dropped upon DSS treatment; however, the decline was significantly more prominent, or even absolute, in the PPIA×Tie-Cre group (Figure 3I,J).

## 3. Discussion

Although the contribution of secreted CypA to the pathogenesis of inflammatory-associated diseases is well elucidated, its potential function as a trigger of such pathologies remains elusive as of today. To decipher the link between elevated systemic levels of CypA and predisposition to developing inflammatory-associated diseases, we generated transgenic mice with the overproduction of human CypA in the blood and induced UC in them.

The PPIA×Tie-Cre mice prior to DSS administration had absolutely normal morphology of the duodenum and colon, with the only differing parameter being the number of goblet cells in the duodenum (*p* < 0.05). After DSS administration, however, the PPIA×Tie-Cre mice developed subtotal crypt destruction, while the control mice retained relatively normal morphology of the colon. We hypothesize that the observed difference may be explained by a primed immunological state formed by elevated systemic levels of pro-inflammatory CypA.

Effectors of both innate (dendritic cells, macrophages, and neutrophils) and adaptive (T and B cells) immunity contribute to UC pathogenesis [28]. Secreted CypA has pleiotropic immunoregulatory effects; particularly, it stimulates maturation and antigen-presenting functions of dendritic cells [29], regulates M1 macrophage polarization [30], induces activation of resting B cells, and enhances functional activity of activated T cells [31]. Furthermore, CypA regulates the production of other pro-inflammatory cytokines [8,9,10]. Hence, elevated blood levels of CypA in PPIA×Tie-Cre mice could trigger activation of immune effectors and promote systemic accumulation of pro-inflammatory factors, thus creating a primed state in these mice and increasing their susceptibility to colitis. Upon DSS administration, CypA could function as a chemoattractant for both innate and adaptive immune effectors [10,11,32], potentially enhancing their infiltration into the inflamed intestine and exacerbating colitis severity, characterized by significant crypt loss and inflammation.

Our results clearly showed that secreted CypA is not only involved in the pathogenesis of UC but also increases the risks of its onset by fostering vulnerability and susceptibility to particular triggers. Since DSS-induced colitis was exploited as a model of inflammatory-associated disease in the studies here, it is anticipated that PPIA×Tie-Cre mice could also be successfully used in mechanistic studies of other pathologies linked to chronic inflammation.

Inflammatory bowel disease (UC and CD) tends to develop early in life, often manifesting during childhood, with the peak of onset between 30 and 40 years of age. As they are long-term chronic conditions, lifelong therapy may be required. Together with a high prevalence, this creates a significant economic burden and impacts quality of life [33,34]. Conventional therapies (pharmacotherapy and surgery) do not fully manage to control the disease. Recent success of specific TNF inhibitors [35] encourages the search for potential novel targets. For this, adequate mouse models are required.

Existing models of IBD can be divided into two groups: genetic models and chemical models. However, genetic models—usually mice with a knockout of an anti-inflammatory gene or overexpression of a pro-inflammatory gene [36]—are too focused on a single aspect of the pathogenesis, while IBDs are almost always multi-factor diseases with no obvious genetic cause. At the same time, chemical models of IBD have their own drawbacks. First, such models are non-physiological from the point of view that UC is a chronic disease, often with a gradual onset, while chemical models better represent acute diseases. Secondly, it is difficult to control the exact dosage of the consumed chemical agent, and animals in experimental groups often have significant variance in the severity of symptoms. This implies that large experimental groups are required [37].

This study describes a novel mouse model of ulcerative colitis that combines a genetic modification approach, specifically, the overexpression of the human *PPIA* gene in the vascular endothelium, and a chemical approach: administration of a low dose of DSS. As a result, the variance in the symptoms among the PPIA×Tie-Cre mice was quite low. Moreover, our model perfectly satisfies the requirement that damage must be localized precisely in the colon, despite CypA circulating in the bloodstream. We believe that our model has several advantages compared to existing models. It better reflects the multifactorial nature of UC and allows for a significant reduction in the number of experimental animals, providing consistent clinical patterns. As the human *PPIA* gene was used, it is suitable for testing CypA-targeted therapies with some considerations.

A critical limitation for translating anti-CypA therapies from preclinical models to human IBD is the potential for species-specific differences in CypA signaling. While CypA is highly conserved across species, functional studies have demonstrated that murine CypA exhibits reduced activity compared to its human ortholog in certain biological contexts [38]. Our model addresses this translational gap by expressing the human protein rather than overexpressing the endogenous one, ensuring that therapeutic antibodies or inhibitors targeting human CypA will encounter their authentic antigen. However, downstream signaling components remain murine, including the CD147 receptor and associated signaling cascades. Although the CypA–CD147 interaction outcomes appear functionally conserved—as evidenced by the efficacy of anti-CD147 antibodies in mouse inflammation models [39]—precise binding affinities and quantitative differences in receptor activation between human CypA/mouse CD147 and human CypA/human CD147 interactions have not been comprehensively characterized. Additionally, immune cell responses to circulating human CypA in the murine system may differ quantitatively from human responses due to species-specific differences in receptor expression levels, post-translational modifications, or co-receptor availability. Hopefully, these responses ought not be of an autoimmune nature, since Tie-Cre is activated early in the development of our model animals. Furthermore, UC in humans is influenced by other complex environmental, microbial, and genetic factors not fully recapitulated here, including the gut microbiome’s role in disease onset and progression [40]. Our model, while colon-specific in its DSS-induced damage, does not incorporate these elements explicitly, which could limit its utility for studying microbiota–immune interactions or personalized therapies. Moreover, the systemic circulation of CypA might not perfectly mirror endogenous CypA dynamics in UC patients, where elevated levels are often a secondary response to inflammation rather than a primary driver [11]. Despite these caveats, the use of human CypA in our model represents a significant advantage for testing many aspects of CypA-neutralizing therapies in both qualitative and quantitative manners.

We hope to develop a precise model of chronic UC based on our mouse strain. By refining repeated DSS administration and combining our model with additional inflammatory stressors, we aim to establish a chronic relapsing–remitting phenotype that more closely mirrors the temporal dynamics of human UC, enabling longitudinal studies of CypA’s role throughout disease progression and therapeutic intervention windows.

## 4. Materials and Methods

### 4.1. Generation of Transgenic Mice

Human *PPIA (hPPIA)* open reading frame (ORF) was amplified with primers Ppia-BshtI-forw 5′-ACCGGTatggtcaaccccaccgtgttc-3′ and Ppia-MluI-rev 5′-ACGCGTttattcgagttgtccacagtcagcaatg-3′ using Platinum SuperFI polymerase (ThermoFisher Scientific, Waltham, MA, USA). The ORF was treated with BshTI and MluI enzymes (ThermoFisher Scientific, Waltham, MA, USA), gel-purified, and cloned into the pKB2 vector intended for random transgenesis described previously [41]. It contained a *LoxP*-flanked STOP-cassette, which attenuates transcription and translation between the CAG promoter and ORF; thus, transgene expression can be activated by Cre recombinase. All the following transgenesis procedures were performed as described previously [42].

### 4.2. Husbandry

All experiments were approved by the Ethics Committee of the Institute of Gene Biology of the Russian Academy of Sciences #28 from 15 November 2024. The animals were kept and treated with tamoxifen if needed, as described previously [43]. The mice were euthanized by cervical dislocation. Blood for CypA protein measurement was collected postmortem from the heart, while blood for qPCR was collected from the retroorbital sinus of mice anesthetized with 200 µL of a Zoletil (Delpharm Tours, Tours, France) and Xyla (Interchemie, Venray, The Netherlands) 2 mg/mL mixture, applied intraperitoneally. Activator strains Tie2-Cre (JAX 8863) [44] and R26-Cre-ER^T2^ (JAX 8463) [45] were purchased previously from Jackson Laboratories. The mice were genotyped for CAG-STOP-*PPIA*, as described previously [41], and for the presence of *Cre*, according to Jackson Laboratories’ protocols.

### 4.3. hPPIA Expression Measurements

*hPPIA* expression at the mRNA level was measured by RT-qPCR, as described earlier [46]. Total RNA was extracted from peripheral blood mononuclear cells with ExtractRNA reagent (Evrogen, Moscow, Russia), treated with DNase I (New England Biolabs, Ipswich, MA, USA), and subjected to reverse transcription. Real-time PCR was performed using qPCR HS-Taq SYBR mix (Evrogen, Moscow, Russia) with primers *Ppia-f* 5′-GCAAGCATGTGGTGTTTGGC-3′ and *Ppia-r* 5′-TTCGAGTTGTCCACAGTCAGC-3′ and the following cycling conditions (40 cycles): 95 °C—20 s, 60 °C—20 s, and 72 °C—30 s. Hypoxanthine phosphoribosyl transferase (*Hprt1*) and beta-actin (*Actb*) were used as housekeeping genes (*Hprt1-f* 5′-CAGCGTCGTGATTAGCGATGA-3′; *Hprt1-r* 5′-GCCACAATGTGATGGCCTCC-3′; *Actb-f* 5′-CGCAGCCACTGTCGAGTC-3′; *Actb-r* 5′-GCCCACGATGGAGGGGAATA-3′). The CypA protein level was measured using a Human Cyclophilin ELISA Kit (EH138RB, Invitrogen, Frederick, MD, USA) according to the manufacturer’s protocols.

### 4.4. Induction of Colitis

DSS application is a common method used to induce colitis in mice [37,47]. Before induction, the mice were tested to ensure they were free of helminths according to the modified G. Baermann method [48]. For colitis induction, drinking water was replaced with a 1%, 3%, or 5% solution of 36–50 kDa DSS (MP Biomedicals, Irvine, CA, USA) for 7 days. After DSS, the mice received drinking water for an additional 7 days, which was followed by another round of DSS when double-round treatment was employed. The disease activity index (DAI) was assessed daily before necropsy based on the following clinical symptoms: weight loss, rectal prolapse, changes in stool consistency, and the presence of blood in the stool, as a sum of scores provided in Table 2 [27]. During necropsy, the length of the large intestine was measured and examined for ulcers and any other macroscopic changes. Histological examination assessed the condition of the lamina propria, the presence of areas of inflammation, the number and morphology of crypts in the large intestine, and the number of Paneth and goblet cells in the duodenum. Five to seven whole random sections were assessed to quantify cell counts and evaluate crypt loss for each subject.

### 4.5. Histology

After 8 h of starvation, the mice were put under non-recovery anesthesia and perfused with 10% Neutral Buffered Formalin (NBF) (Medix, Taganrog, Russia) in PBS pH 7.4. Duodenum and intestine specimens were further fixed in 10% NBF for 24 h, washed in water, and processed through dehydrating solution, intermediate media, mineral oil, and paraffin universal medium (Medix, Taganrog, Russia) according to the manufacturer’s protocol. Paraffin-embedded 5 µm thick sections were stained with hematoxylin–eosin and periodic acid Schiff reaction. All samples were examined, and images were acquired using a Nikon ECLIPSE Ti microscope (Nikon Corporation, Tokyo, Japan).

### 4.6. Statistical Data Analysis

All statistical analyses and plotting were carried out in GraphPad Prism 10.4.0 software. The normality of sample distributions was assessed using the Shapiro–Wilk test. For multiple comparisons of cell counts and colon lengths, two-way and one-way ANOVA analyses with Šídák’s post hoc test were used, respectively. For CypA protein level comparisons, the Kruskal–Wallis test with Dunn’s post hoc test was used. Differences with *p* < 0.05 (*), *p* < 0.021 (**), *p* < 0.002 (***), and *p* < 0.0001 (****) values were considered statistically significant.

## Data Availability

Data are contained within this article.

## Abbreviations

The following abbreviations are used in this manuscript:

CypA	Cyclophilin A
CAG	Cytomegalovirus–actin–globin
IBD	Inflammatory bowel disease
CD	Crohn’s disease
UC	Ulcerative colitis
ORF	Open reading frame
NBF	Neutral buffered formalin
DSS	Dextran sulphate sodium
